# Emerging roles of mesenchymal stem cell therapy in patients with critical limb ischemia

**DOI:** 10.1186/s13287-022-03148-9

**Published:** 2022-09-06

**Authors:** Zeinab Shirbaghaee, Mohammad Hassani, Saeed Heidari Keshel, Masoud Soleimani

**Affiliations:** 1 Medical Nanotechnology and Tissue Engineering Research Center, Shahid Beheshti University of Medical Sciences, Tehran, Iran; 2 Department of Tissue Engineering and Applied Cell Science, School of Advanced Technologies in Medicine, Shahid Beheshti University of Medical Sciences, Tehran, Iran; 3 Department of Vascular and Endovascular Surgery, Ayatollah Taleghani Hospital Research Development Committee, Shahid Beheshti University of Medical Sciences, Tehran, Iran; 4 Applied Cell Science and Hematology Department, Faculty of Medical Science, Tarbiat Modares University, Tehran, Iran

**Keywords:** Critical limb ischemia, Angiogenesis, Cell therapy, Mesenchymal stem cells, Clinical trials, Hindlimb ischemia, Peripheral arterial disease

## Abstract

Critical limb ischemia (CLI), the terminal stage of peripheral arterial disease (PAD), is characterized by an extremely high risk of amputation and vascular issues, resulting in severe morbidity and mortality. In patients with severe limb ischemia with no alternative therapy options, such as endovascular angioplasty or bypass surgery, therapeutic angiogenesis utilizing cell-based therapies is vital for increasing blood flow to ischemic regions. Mesenchymal stem cells (MSCs) are currently considered one of the most encouraging cells as a regenerative alternative for the surgical treatment of CLI, including restoring tissue function and repairing ischemic tissue via immunomodulation and angiogenesis. The regenerative treatments for limb ischemia based on MSC therapy are still considered experimental. Despite recent advances in preclinical and clinical research studies, it is not recommended for regular clinical use. In this study, we review the immunomodulatory features of MSC besides the current understanding of different sources of MSC in the angiogenic treatment of CLI subjects and their potential applications as therapeutic agents. Specifically, this paper concentrates on the most current clinical application issues, and several recommendations are provided to improve the efficacy of cell therapy for CLI patients.

## Introduction

Peripheral arterial disease (PAD) is chronic vascular disease that causes impaired circulation to the lower extremities [1, 2]. A subset of PAD patients has critical limb ischemia (CLI), representing the end-stage PAD [3] described by intractable rest pain, tissue ulcers, necrosis or gangrene, and finally, death in the context of definitive hemodynamic indicators of vascular dysfunction [4]. Critical limb ischemia caused by tissue hypoxia accompanied by impaired life quality, increased mortality and morbidity, and significant socioeconomic and social impacts [3, 5]. CLI occurs in up to 1 in 10 people with PAD, and 5–10% of patients with intermittent claudication (IC) transform CLI within 5 years [6]. The disease's prevalence rises with growing rates of diabetes, nicotine use, hypertension, and hypercholesterolemia [3]. Endovascular therapy, surgical bypass, and limb amputation are the only available therapies for subjects with arterial disease of the lower limbs and related disorders [7]. 20–30% of patients with CLI are ineligible for revascularization, or the surgery has failed due to distal flow impairment [8]. The Food and Drug Administration (FDA) has not yet approved any additional effective CLI therapies, underlining the essential requirement to explore alternative treatment options to recover the blood flow and to enhance the grade of healthcare services for life-threatening conditions [9, 10]. As extensive gene therapy trials studies have failed, even though there is continuing attention to assessing HGF [9], and there are concerns regarding cell therapy, such therapy has not yet been approved for clinical application. Trials using growth factors singly cannot provide the fundamental factors that patients with CLI require [11]. Compared to approaches based on proteins or genes, cell-based treatments appear to be more effective because of their natural vasculogenic characteristics and their paracrine impact [12]. Angiogenic cells may directly contribute to the development of new blood vessels and produce endogenous growth factors that stimulate vascular expansion [13, 14]. MSCs (mesenchymal stem cells) are thought to be one of the most beneficial cells as a regenerative therapeutic option for CLI due to their unique biological features [15]. MSCs can release angiogenic factors and undergo endothelial differentiation. Therefore, they can induce angiogenesis, restore blood circulation to ischemic sites, and promote tissue regeneration and functional recovery [7]. MSCs and endothelial cells (ECs) engage in a complicated "cross-talk" MSCs stimulate the growth and relocation of ECs to initiate the early phases of angiogenesis and lessen the permeability of the EC monolayer. In MSCs and ECs cocultures, MSCs improve dose-dependently the durability of preexisting blood vessels [15, 16]. Several studies have confirmed that one of the crucial activities of MSCs is to release bioactive substances relevant to the "niche" in which they are embedded. As a result, under resting and inflammatory situations, MSCs create an extensive range of pro- and anti-inflammatory cytokines, growth factors, and prostaglandins, which are transiently reproduced by the secretome [17, 18]. MSCs have low MHC class *I* expression levels and lack MHC-class II and costimulatory markers, including CD80, CD86, CD40, and CD40L. Nonetheless, MSCs express surface molecules such as VCAM-1, ICAM-2, and LFA-3, which are critical for T cell interaction with the thymic epithelium [19]. Whereas MSCs remain inactive, exhibiting anti-apoptotic properties and contributing to homeostasis, they initiate to practice their immunomodulatory attributes in an inflammatory environment, inhibiting effector cell proliferation and cytokine generation. MSCs could inhibit several immune cell functions. They can therefore be utilized as an allogeneic cell source [20].

Here, we detail the current understanding of the role of MSC-mediated angiogenesis in CLI and their potential applications as therapeutic agents. We further define the most recent challenges in clinical application, and some provide recommendations to enhance the prospective effectiveness of cell treatment for CLI patients. The fact that the regenerative treatments of limb ischemia based on cell therapy are still considered experimental, and despite recent advances in preclinical and clinical research studies, it is not recommended for regular clinical use [3] proposes that there are insufficient data on the feasibility of specific cell types and their application strategies.

### Mesenchymal stem cells and pathophysiological mechanisms of ischemic diseases

Most ischemic disorders are caused by atherosclerosis that is a chronic inflammatory disease of the arteries [21]. Atherosclerosis is attributed to impairment and dysfunction of ECs lining the luminal wall of arteries, smooth muscle cells (SMCs), leukocytes, "foam cells," accumulation of lipids, and aggregated platelets at the luminal side of the artery, and plaque development. Both platelets and macrophages produce matrix metalloproteinase (MMPs) to destroy a blood vessel's collagenous extracellular matrix (ECM) [22, 23]. SMCs transit from the tunica media and adventitia (the outer layers of the artery wall) to the tunica intima of the arterial wall to enhance the collagen synthesis rate to counteract MMP-mediated ECM degeneration [24]. Nevertheless, macrophages typically bring about unsatisfactory remodeling because they release cytokines (e.g., TNFα, IL-1, and IL-6) to induce SMCs apoptosis [25]. When the frequency of collagen creation is inadequate to counterbalance the rate of ECM degradation, atheromatous plaques will occur with a fibrous collagenous cap [26]. At this point, MSC therapy may decrease immune cell activities (MMP activity and pro-inflammatory cytokine release) and restore collagen balance, indicating that MSCs can be used to treat atherosclerosis in order to prevent tissue ischemia [27].

### Mechanisms of MSC-based immunomodulation

MSCs can modulate their immunomodulatory actions based on the amounts of soluble factors such as GM-CSF or INF-g, IL-8, and MIF, along with mitochondrial translocation [28] and the microenvironment of inflammatory diseases in general. For instance, in graft-versus-host disease (GvHD), MSCs can restrict Th1 and Th17 polarization while promoting Th2 polarization. MSCs can also decrease Th2-dominant allergies by suppressing the production of IL-4 and IL-13. In acute or chronic inflammatory situations, MSCs suppress the immune system or assist in the fibrotic process. MSCs are a versatile and practical technique for treating a wide variety of disorders due to their immunomodulatory properties [29]. Acute inflammation causes M1 macrophage polarization by Th1 cytokines, which proceeds to MSC licensing via released TNF-α and IFN-γ. Under chronic inflammation, M2 macrophages get polarized and produce IL-10 and 6, which alternatively license MSCs [30].

**Fig. 1:**
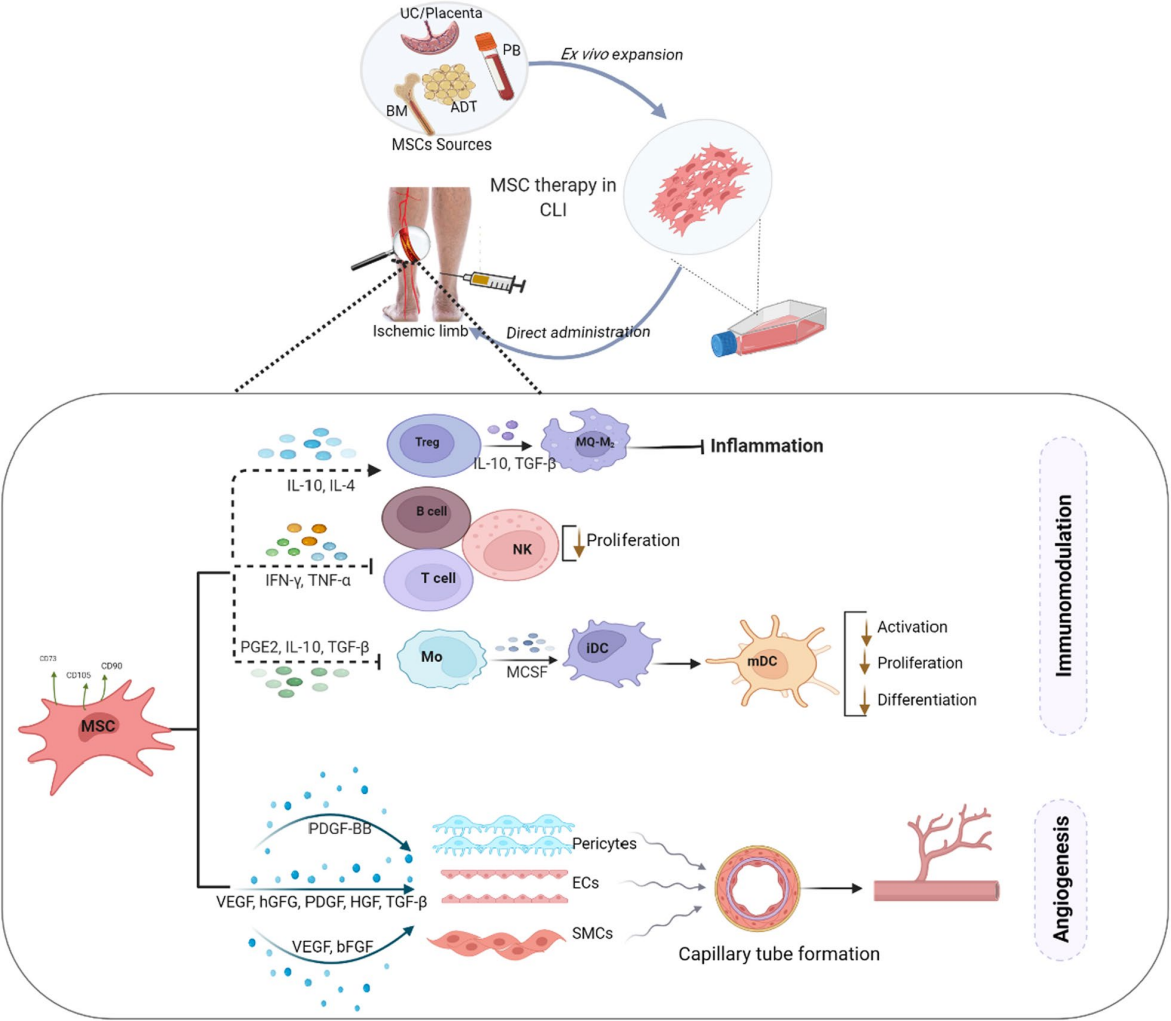
Major sources of mesenchymal stem cells (MSCs) and potential mechanisms of MSCs therapy in limb ischemia. MSCs are isolable from several sources. They restore tissue function and repair ischemic tissue via immunomodulation and angiogenesis. MSCs suppress inflammation and promote immunomodulation by secreting immunomodulatory cytokines, which stimulate the induction of M2 macrophages and increase the number of circulating regulatory T cells, resulting in an increase in interleukin IL-10 and resolution of inflammation. Additionally, MSCs release factors that promote angiogenesis directly. The possible mechanism by which MSCs mediate angiogenesis via direct dedifferentiation or through paracrine effects on effector cells such as smooth muscle cells, endothelial cells, and pericytes in the formation of mature vessels. *ADT* adipose tissue, *BM* bone marrow, *CLI* critical limb ischemia, *ECs* endothelial cells, *iDC* immature dendritic cell, *mDC* mature dendritic cell, *MO* monocyte, *MQ-M*_*2*_ macrophage M_2_, *MSC* mesenchymal stem cells, *NK* natural killer cells, *PB* peripheral blood, *SMCs* smooth muscle cells, *T-reg* regulatory T cell, *UCB* umbilical cord blood

MSCs have a unique ability to evade and modulate the immune system, well-documented along with several MSC tissue sources [31]. MSCs' immunomodulatory ability results in an immune tolerant phenotype. They express low amounts of MHC Class I surface antigens and no MHC Class II antigens except if inflammatory signaling is initiated [32, 33]. MSCs also produce HLA-G, which is typically associated with the development of immunological tolerance at the maternal–fetal junction, and express the coinhibitory molecules PD-L1 (B7-H) and VTCN1 (B7-H4) to enhance the immune evasion [34]. MSCs further restrict the production and pro-inflammatory capabilities of CD4+ T helper cells (Th1 and Th17), simultaneously stimulating the proliferation of regulatory T cells and decreasing the development, cytokine production, and cytotoxic activities of pro-inflammatory CD8+ T cells, using their inhibitory capacities [35]. Li et al. conducted a clinical trial to assess the distribution of Tregs/Th17/Th1 cells in diabetes patients before and after implantation of hUCB-MSCs. The CD4^+^ CD25 FoxP3^+^ Treg/Th1 and CD4^+^ CD25 FoxP3^+^ Treg/Th17 cell proportions were considerably enhanced 4 weeks after hUCB-MSCs administration, but the Th17/Th1 cell ratio remained constant [36] (Fig. 1).

MSCs lower monocytes/macrophages' phagocytic and antigen-presenting capabilities and immunosuppressive molecule expressions such as interleukin IL-10 and PD-L1 [35]. MSC can prevent the development of CD14+ CD1a progenitors into dermal DC via IL-10, PGE2, and downstream JAK/STAT signaling without influencing the formation of CD1a+ Langerhans cells. During LPS-mediated maturation of iDC (immature DC) into mDC (mature DC), TSG-6 (TNF-stimulating gene-6), an MSC-produced, inhibits MAPK and NF-kB signaling activity [37]. As a result, they excel at inhibiting DCs and macrophages' maturation and function and their capability to produce pro-inflammatory cytokines and inducing robust T cell responses, while inducing a transition into anti-inflammatory M2 macrophages and regulatory DCs [35, 37]. Enhancement of anti-inflammatory cytokines, including IL-10/Arginase-1, and reductions of pro-inflammatory TNF-α, IL-1b, and IL-12 production is associated with MSC-induced M2 polarization [25, 38]. Decreased expression of costimulatory molecules in M2 macrophages, such as CD40, CD40L, CD80 (B7-1), and CD86 (B7-2), resulting in significant immunosuppression and encouraging the beneficial outcomes of Treatment using MSCs [39, 40]. MSCs also stimulate the differentiation of regulatory B cells that produce IL-10 by inhibiting B-cell development, growth, and antibody generation [35]. They can suppress the production and cytotoxic activity of NK cells. There is proof that the persistence of MSCs considerably inhibits IL-2/15-induced NK cell proliferation, IFN production, perforin/granzyme levels, and cytotoxicity [41–43]. In addition, the expression of surface receptors implicated in NK cell stimulation and death of target cells, such as NKp30, NKp44, and NKG2D, was downregulated. Furthermore, MSC-produced immunosuppressive secretors such as IDO, TGF-β, PGE-2, and HLA-G participate in MSC-mediated NK cell suppression [44].

Several studies have revealed contradictory results concerning the impacts of MSCs on NK cell proliferation, with some claiming inhibitory effects and others describing stimulatory ones [43, 45–47]. These disparate outcomes are expected to be highly influenced by the dose of the MSCs and their origins, cell-cell interaction, injection routes and durations, cell content, as well as the recipient's immunological condition prior to and following transplantation [48]. Likewise, inter-donor variations, as well as the origin of MSCs, may significantly influence the expression of ligands for NKG2D (MICA/B and ULBPs), CD244 (CD48), DNAM-1 (CD112, CD155), and NCR (NKp46-Fc, NKp44-Fc, and NKp30-Fc) receptors [43, 49, 50] and thus modify NK cell interactions and operations. A decrease in NK cell activity in MSCs may be due to a modified expression of CD226. Bone marrow-stromal cell-NK cell interaction has been found to cause the activation of NK cells irrespective of CD226 [50, 51]. These disparate results demonstrate that both cell types' functions depend on their surroundings. Pro-inflammatory cytokines from ongoing immune responses, such as those utilized to activate NK cells, may dramatically modify their receptor pattern and characteristics [52].

Moreover, MSCs express TLRs 2, 3, 4, 7, and 9 dynamically, influencing their pro or anti-inflammatory characteristics based on the microenvironmental background [48]. TLR-specific activation of MSCs appears to dictate the fate of their unique immunomodulatory roles. TLR4 stimulation of MSCs results in the pro-inflammatory MSC1 phenotype, while TLR3 innervation results in the immunomodulatory MSC2 phenotype [53]. MSC1 produces more pro-inflammatory cytokines and chemokines, such as IL-6 and IL-8 than MSC2, which produces more immunosuppressive mediators, such as CCL5 and IP-10. Numerous factors, including the origin tissue, species of source, and environmental factors, account for the diversity of responses found after TLR stimulation in MSCs [54].

MSCs mitochondrial transfer to immune cells is another method of activating immunomodulatory pathways and tissue repair and regeneration. When cells are stressed or injured, they start releasing and delivering mitochondria to MSCs, which has been demonstrated to trigger anti-apoptotic activity and mitochondrial biogenesis in MSCs. MSCs exposed to mitochondria from injured cells, which have pro-regenerative and cytoprotective capabilities, could be used to precondition MSCs for increased and targeted therapeutic efficacy. Nonetheless, the methods of mitochondrial translocation via nanotubes, GAP junctions, cell fusion, uptake or microvesicles, and the frequency, efficacy, and donor cells, must be investigated further before therapeutic strategies based on mitochondrial transplantation may be developed [28].

Moreover, innovative techniques such as chemical preconditioning and hypoxia pre-treatment can substantially improve MSC immunosuppressive potency [29]. Preconditioned umbilical cord-derived MSCs have been demonstrated to be more effective in treating mice model of hindlimb ischemia [55]. Hypoxia may also improve MSCs' supporting role on endothelial progenitors, as evidenced by diabetic rats with hindlimb ischemia [56]. IFN-γ and TNF-α have been shown to stimulate the production of immunomodulatory factors by MSCs; however, these effects are temporary [29]. TNF-primed MSCs have greater immunomodulatory and tissue-repair capacity than non-primed controls, as shown by higher secretion of immunomodulatory molecules such as PGE2, sTNFR, and TSG-6; chemokines such as IL-8, CXCL5, and CXCL6; proangiogenic growth factors such as FGF2, VEGF, or IL-8; along with enhanced tunneling nanotube (TNT) production allowing mitochondria translocation to reduce damage via the TNFR1 or TNFR2 signaling pathway. Based on the kind and stage of disease, TNFR1 signaling can have two different consequences on MSC-based therapy for autoimmune or inflammatory diseases. Although TNFR1 has a dual effect on MSC efficacy, TNFR2-mediated signaling boosts MSC efficacy overall [57]. IFN-γ is another cytokine that has an impact on MSCs. When this cytokine is produced by CD4+ helper T cells and cytotoxic CD8 T lymphocytes, MSCs become "licensed" with T cell inhibitory characteristics. Responses of MSCs to IFN-γ appear to be species-specific, with human MSCs upregulating IDO and mouse MSCs upregulating iNOS [58]. According to Li et al., MSC-mediated immunomodulation can be switched on or off depending on the level of NO generation. When inducible nitric oxide synthase (iNOS) is suppressed, MSCs prefer T cell proliferation instead of immunosuppression. INOS/IDO levels strongly influence the pathophysiological functions of MSCs [59]. MSCs exposed to IFN-γ can also generate suppressive costimulatory molecules such as B7 family coregulatory molecules B7-H1, which interact with CD4+ cells, inhibit cell growth, and make T cells anergic. Although changes in donor sources or culture conditions may potentially play a role in changing the influence of IFN-γ on MSC efficacy, it is interesting to note that Interferon alters MSC effectiveness in a dose-dependent manner that can trigger the immunosuppressive MSC impacts. IFN-γ can produce MHC classes I and II on MSCs, making them immunogenic [54]. According to several research, low amounts of IFN-γ and TNF-α, as well as long-term exposure to these cytokines, transform MSC from an immunosuppressive to a pro-inflammatory state. Thus, these inflammatory cytokines can affect the effects of MSCs on Tregs, thereby modifying their potency against autoimmune and inflammatory disorders [57]. Finally, TGF-β has been demonstrated to inhibit MSC anti-inflammatory capabilities by lowering iNOS expression, implying a feedback loop in the microenvironment wherein TGF activation may enhance inflammation resolution and/or tissue regeneration [54].

### Major signaling pathways regulating mesenchymal stem cells angiogenic properties

Different studies that indicate the MSC involvement in maintaining in vivo structures of neovessels have identified several molecular pathways [60, 61].

The Wnt pathways have been found to have an essential role in the adjustment of MSC differentiation, proliferation, and migration. WNT4 activation in MSC has been shown to increase blood flow. frizzled-related protein-1, a Wnt modulator secreted by MSCs, promotes angiogenesis by increasing MSC integration into neovessels, implying that specific molecular targets are responsible for MSC engraftment into the vasculature [62, 63]. DKK1 is a Wnt antagonist that inhibits the Wnt pathway, which suppresses pericyte growth and migration through the Wnt coreceptor (LRP6). The canonical Wnt pathway has also been shown to be vital in the transformation of c-KIT+ progenitor cells into SMCs [64].

TGF-β signaling regulates MSCs differentiation into pericytes and SMPCs. TGF-β signaling promotes MSC proliferation and MSC differentiation to SMCs through Smad3 and Smad2/3-mediated pathways [64, 65]. BMP4-mediated SMAD1/5/8 phosphorylation also promotes the differentiation of MSCs into Ecs [66]. TGF-β regulates the Notch ligand JAG1 expression. It was shown that Notch signaling triggered by JAG1 stimulates MSC differentiation into SMCs. MSC differentiation into SMCs is supported by Notch signaling in combination with the Hedgehog pathway [37, 67].

Another intracellular signaling pathway involved in the survival of MSCs includes the activity of the PI3K—protein kinase B (serine-threonine protein kinase Akt) pathway. Overexpression of Akt1 in MSCs helps to improve the survival of MSCs following transplantation into rats' hearts. MSC overexpressing Akt made more of the target proteins downstream of Akt, like the anti-apoptotic protein Bcl-2. On the contrary, the pro-apoptotic protein Bax was reduced [68].

### Evaluation criteria of functional and clinical changes in MSC therapy trials

While most studies on cell therapy have not yet demonstrated specific adverse events (AEs) and the number of patients in particular trials tends to be relatively small, the safety endpoint of cell therapy is rarely addressed in detail in the published research. Safety evaluations include monitoring and documentation of AEs [69, 70]. Several adverse events have been observed in individuals treated with stem cell transplantation, including MACEs, death, anemia, hemorrhage, infection, pain, injection-induced rhabdomyolysis, renal injury, and malignancy [71]. Along with the routine safety laboratory measures, the immunological response to the cell therapy was considered AEs identified by measuring pro-inflammatory cytokines such as IL-2, IFN-γ, and TNF-α and determining the lymphocyte profile (CD4, CD8, and CD25) before and following MSCs administration [69].

CLI therapy aims to reduce disease progression, improve limb salvage, and ameliorate symptoms and quality of life (QoL). Various endpoints are utilized in clinical trials to indicate the potency of therapy in accomplishing one or more of these goals. The efficacy analysis was separated into functional and clinical endpoints. Functional endpoints consist of clinical classification of disease severity (such as Rutherford or Fontaine class), wound healing, change in pain score, and QoL, as well as hemodynamic measures such as ankle-brachial index (ABI) or transcutaneous oxygen pressure (TcPO2), which are indicators of enhanced limb perfusion and angiographic evidence of new blood vessel development [71] (Table 1).

**Table 1:** Functional and clinical endpoints in MSC therapy of critical limb ischemia (CLI) trials

SAFETY	Adverse events (AEs)		Death
			MACEs
			Anemia
			Bleeding
			Pain
			Fever
			Infection
			Transient allergic reactions
			Injection-induced rhabdomyolysis
			Kidney injury
			Gangrene
			Proliferative retinopathy
			Unregulated Angiogenesis: Arterio-Venous (A-V) Malformations and Retinopathy
			GVHD
			Cancer
	Immunogenicity		Plasma cytokine levels (TNF-a, IL-6, and IL-1b)
			Lymphocyte profile (CD4, CD8 and CD25)
EFFICCY	Functional endpoints	Subjective perfusion endpoints	Ischemia severity according to Rutherford or Fontaine
			Ulcer healing
			Pain score
			Quality of life (SF-36 or VascuQoL)
			Exercise treadmill test (PWD)
		Objective perfusion endpoints	ABI, TcpO2, TBI
			Angiography
	Clinical endpoints	Death rate	
		Amputation rate	
		Major amputation of the index leg	
		Amputation-free survival	
		Time to death or amputation of the index leg	

*ABI* ankle-brachial index, *AEs* adverse events, *GVHD* graft-versus-host disease, *MACEs* major adverse cardiovascular events, *MWD* maximal walking distance, *PWD* pain-free walking distance, *TBI* toe-brachial index, *TcPO*_*2*_ transcutaneous oxygen pressure

Classification methods for CLI severity were devised to estimate amputation probability and treatment outcomes. Historically, the Fontaine and Rutherford classification systems categorized patients based on clinical criteria alone or in conjunction with objective hemodynamic evidence [72].

Ischemic rest pain, as measured by the Visual Analog Scale (VAS) or other pain intensity scales like the Numeric Rating Scale (NRS) and Wong–Baker faces pain rating scale (WBFPRS), typically manifests as a persistent burning feeling or numbness in the foot mainly in the absence of movement [73]. Therefore, a reduction in resting pain of more than 50 percent at different times is considered an improvement [12].

Studies assessing the quality of life in CLI patients have utilized a mixture of standard quality of life surveys (such as SF-36) and disease-specific questionnaires (i.e., VascuQoL). Disease-specific quality of life measures are ideal for CLI populations because they address the patient's specific restrictions, making them more able to identify significant clinical changes in health conditions regarding the progression of the disease or treatment [2]. Another functional outcome is improved wound healing that an independent physician evaluates and evidences by photography at the end of the follow-up period [10].

The most commonly used test, the ankle-brachial index (ABI), measures the proportion of maximum arm blood pressure to maximum ankle blood pressure in the affected limb [73]. The reported data reveal that patients undergoing cell therapy have an improved state of the afflicted extremity in all criteria. For instance, Benoit et al. accomplished a meta-analysis that exhibited increased ABI values in 63.2% of patients, significantly increased TcPO2 in 76.9% of patients, pain relief in nearly 90%, and claudication interval extension in 89.5% of patients [3]. Angiography is a kind of functional endpoint that utilizes mean percentage vascular flow change from angiographic techniques, including duplex ultrasonography (DUS), computed tomography angiography (CTA), and magnetic resonance angiography (MRA), to evaluate the target limb's vascularity [72].

Clinical endpoints are related directly to prognosis and survival, which are outcomes of considerable interest to patients and doctors. Therefore, cell therapy must show efficacy in terms of amputation and death to become a generally accepted treatment [71]. Amputation is correlated with poor overall survival; Amputation is the only choice when ischemic tissues are exposed to uncontrollable infections and when surgical or non-surgical techniques cannot repair rest pain or tissue loss [10].

Patients with CLI have high death rates due to vascular impairment and comorbidities like diabetes and renal disease. Because mortality rates increase over time in trials with longer follow-ups, the impact of innovative treatment on mortality may be hidden by deaths from other reasons. This characteristic makes it difficult to compare mortality rates between groups with various follow-up durations. In the context of clinical trials, it is essential to evaluate all-cause mortality to identify any elevated risks offered by innovative therapies [71].

### Mesenchymal stem cells and therapeutic applications in CLI patient

MSCs are a kind of stem cell that could differentiate into mesenchymal lineages such as osteoblasts, chondrocytes, myoblasts, and adipocytes. CD105 (SH2), CD73 (SH3), and CD90 are expressed on the cell surface of human MSCs. Because of their multipotency, MSCs have been considered a promising alternative for cell therapy targeting ischemic cardiovascular disorders [74]. They restore blood circulation to the ischemic limbs via angiogenesis, thereby using endothelial proliferation and paracrine pathways to prevent limb amputation [75]. Autologous, allogeneic, and xenogeneic MSCs generated from diverse sources such as bone marrow, umbilical cord blood, Wharton's jelly, amniotic fluid, adipose tissue, and the placenta have been proven to be advantageous in preclinical investigations using mouse and rat models of ischemia of the lower extremities [1] (Table 2; Fig. 1). Following that, the encouraging results of preclinical research prompted several clinical trials to exhibit the safety and effectiveness of MSCs generated from diverse sources for the treatment of subjects with CLI (Table 3). Because many of these studies are currently recruiting subjects, their results are not yet accessible. Detailed information about clinical studies is available at www.clinicaltrials.gov.

- Bone marrow-derived mesenchymal stem cells (BM-MSC)

**Table 2:** Biological properties of tissue-derived MSCs

MSC source	Tissue	Stem cells derived	Specific marker	Type	Trilineage potential	Secretome	Immunomodulation	Advantages	Disadvantages
Adult	Bone marrow	BM-MSCs	CD73, CD90, CD105, CD146/MCAM, CD271, MSCA-1, CD29, CD44, STRO-1, OCT4, NANOG, SSEA4	Autologous/allogeneic	Osteogenic and chondrogenic	IL-7, IL-12, MMP-1, MMP-3 miR-486, miR-10a, miR-10b, miR-191, miR-222	Inhibition of T cells, IDO	Cost-effective procedure, ability to use autologously, lack (autologous) and low risk (allogenic) of immune rejection	Invasive and painful harvest, risk of infection, limited by the donor’s age, sex and physical condition, slow proliferation rates, low quantities, earlier appearance of senescence
Adult	Adipose tissues: fat, liposuction	Ad-MSCs	DPP4/CD26, PDGFRa, CD29, CD34, CD36, SCA1, CD55, THY1/C90, CD24, BMP7, PI16, WNT2, ANXA3, coagulation factor III or tissue factor (TF/CD142) (268),	Autologous/allogeneic	Higher adipogenic potential	PDGF-BB, MCP1, SDF-1, TGF-β1, VEGF ANG, HIF-α, MMP9, Bcl2, VCAM miR-143, miR-10b, miR-486, miR-22, miR-211	IDO, PDL-1, IL-10, inhibition of T cells	Cost-effective procedure, less invasive techniques and painful, lack (autologous) and low risk (allogenic) of immune rejection, easily accessible, more resistant to senescence, high yield, easy to cryopreserve	Limited by the donor’s age, sex and physical condition
Newborn	Extraembryonic tissues: umbilical cord Wharton’s jelly Amniotic membrane amniotic fluid Placenta	WJ-MSCs, Am-MSCs, YS-MSCs, UC-MSCs, UCB-MSCs, AF-MSCs	CD146, CD10, CD49d (integrin a4), CD54 (ICAM1), CD 491 (240), CD200, and PDL2, SSEA4, OCT4,	Allogeneic	Conflictive result: P-MSC: higher osteogenic (247, 259) and chondrogenic potential UCB-MSC: higher osteogenic and adipogenic potential	VEGF, HGF, IL-1 RA, IFN-α, IL-6, IL-8, TGF-β2, PDGF-AA, G-CSF3 MiR-21, miR-23a, miR-125b, miR-145	PDL-1, PDL-2, CD10, CD146, CD49d, IDO, IL-1β, LIF, TNF-**2,** inhibition of T cells	Cost-effective, medical waste, procedure, not limited by the donor’s age and physical condition, high abilities to induce angiogenic phenotypes, lowest expression of HLA antigens, high proliferation rates, no immune reactions	Smaller yields
Adult	MSC sources	iPSC-MSC	CD73, CD90, and CD105, CD29, CD44, CD146	Autologous/allogeneic	Less effectively along the adipogenic, osteogenic, or chondrogenic	IL-1-, TSG6, VCAM1, TGFB1,	Nanog, Oct4, and Msx1, HIF-1α VEGFA), VEGFB, placental growth factor (PGF), bFGF, TGFB1,	No ethical concerns, unlimited cell number, fast proliferation, Longer life span, Lower variation	Potential for teratoma and teratocarcinoma, no clinical data, Lower differentiation potential, impaired immunosuppression, immature differentiation potential

MSCs can be isolated from adult tissue sources such as adipose (Ad) and bone marrow (BM), as well as perinatal and/or birth-associated tissues, including amniotic liquid (AM), placenta (P) or umbilical cord (UC) tissues. Tissue of origin have shown to impact the biological properties of MSCs

*WJ-MSCs* Wharton's jelly-derived mesenchymal stem, *Am-MSCs* amniotic membrane-derived mesenchymal stem, *YS-MSCs* yolk sac-derived mesenchymal stem cells, *UC-MSCs* umbilical cord-derived mesenchymal stem cells, *UCB-MSCs* umbilical cord blood-derived mesenchymal stem, *AF-MSCs* amniotic fluid-derived mesenchymal stem

**Table 3:** Classification of most important MSC therapy clinical trials for CLI

NO	Trial ID	Phase/study condition	MSC source	Disease stage	Administration	Administration route	Endpoints	Follow-up (month)	Patient enrollment
1	NCT00468000	II/completed	Ixmyelocel-T (BM-MNCs and BM-MSCs)	NA	35 × 10^6^, 295 × 10^6^	IM	AFS, ABI, tcpO_2_, AR, UH, VAS	12	72 (48/24)
2	NCT00518401	I/completed	MESENDO (BM-MNCs and BM-MSCs)	NA	20 × 10^6^, 40 × 10^6^	IM	AEs	6	10
3	NCT00721006	II/completed	MESENDO (BM-MNCs and BM-MSCs)	Rutherford 4–6	9 × 10^6^, 18 × 10^6^	IM	PWD, ABI	4	26
4	NCT00883870	I/II/completed	Stempeucel(R) (allogenic BM-MSCs)	Rutherford 4–6	200 × 10^6^	IM	AEs, ABI, AR, NRS, UH	6	20 (10/10)
5	NCT00955669	I/completed	BM-MSCs or MNCs	Fontaine 5	9.3 ± 1.1 × 10^8^ BM, MSC/9.6 ± 1.1 × 10^8^ MNC	IM	AEs, UH, PWD, ABI, tcpO_2_, ASM	6	40
6	NCT01065337	II/completed	BM-MNCs and BM-MSCs	Fontaine 3–5	200 × 10^6^, 300 × 10^6^	IM	ABI, TcPO_2_, UH, ILP	12	30
7	NCT01351610	I/II/completed	MSC_Apceth (BM-MSCs)	Rutherford ≥ 4	NA	IA	AEs, VAS	12	25
8	NCT01484574	II/completed	Stempeucel(R) (allogenic BM-MSCs)	Rutherford 3–5	NA	IM	UH, NRS, PWD, AFS, ABI, tcpO_2,_ RA	24	90
9	NCT01456819	II/unknown	BM-MNCs or BM-MSCs	NA	NA	IM	UH, VAS, DSA, ETT	12	50
10	NCT01483898	III/completed	Ixmyelocel-T (BM-MNCs and BM-MSCs)	Rutherford 5	35 × 10^6^, 295 × 10^6^	IM	AFS, UH, MACE	18	41
11	NCT02336646	I/completed	BM-MSCs	Rutherford 4–6	NA	IM	AEs, ABI, tcpO_2_, WBFRS, TWD	6	18
12	NCT02685098	I/II/recruiting	BM-MSCs	Rutherford 2–4	NA	IM	AEs, TcPO_2_, ABI, ICA	24	16
13	NCT03042572	II, III/N/A	BM-MSCs	Rutherford class 4–5	150 × 10^6^	IM	VAS, ABI, TBI, PWD, UH	6	66
14	NCT03455335	I_b_/completed	BM-MSCs	Rutherford 4, 5	20 × 10^6^, 40 × 10^6^	IM	AEs, AR, ABI, TcPO_2_, NRS, UH	12	12
15	TRI/2018/02/011839	IV/completed	Stempeucel(R) (allogenic BM-MSCs)	Rutherford 5–6	2 × 10^6^ cells/kg	IM	AEs, ABI, UH, VAS	12	50
16	NCT01257776	I, II/completed	Ad-MSCs or MNCs	Rutherford 2–4	0.5 × 10^6^, 1 × 10^6^ kg/ml	IA	AEs, UH, tcpO_2_, and ABI	12	36
17	NCT01211028	I, II/completed	Ad-MSCs	Rutherford 2–6	100 × 10^6^	IM	AEs, UH, NRS, VAS, tcpO_2_, ABI	6	13
18	NCT01302015	NA/completed	RNL-Vascostem^®^ (Ad-MSCs)	Rutherford 4–6	300 × 10^6^	IM	AEs, UH, ABI, ETT, DSA, AR, WBFRS	6	15
19	NCT01745744	II/completed	Ad-MSCs	Rutherford 2–4	0.5 × 10^6^, 1 × 10^6^ cell/kg	IA	AEs, ABI, tcpO_2,_ ulcer size, MWD,	12	33
20	NCT01663376	NA/completed	Ad-MSCs	Rutherford 4–6	100 × 10^6^, 300 × 10^6^	IM	AEs, ABI, DSA, thermography, WBFRS, ETT	12	20
21	NCT01824069	I_b_/completed	Ad-MSCs	Rutherford 4–5	1 × 10^6^ cells/kg	IM	AEs, ABI, AR, ILP	12	7
22	NCT02145897	I, II/unknown	Ad-MSCs	Rutherford 4–5	1 × 10^6^ cells/kg	IM/IV	AEs, ABI, tcpO_2_, UH	9	60
23	NCT03968198	II/recruiting	Ad-MSCs	NA	90 × 10^6^	IM	AFS, ABI, tcpO_2_, UH	6	43
24	NCT04466007	II/recruiting	Ad-MSCs	Rutherford 4–5	1 × 10^6^ cells/Kg, 2 × 10^6^ cells/Kg	IM	AEs, vascularization, Rutherford–Becker scale, ABI, AR	12	90
25	NCT04661644	I, II_a_/recruiting	Ad-MSCs	Rutherford 4–6	1 × 10^7^ cells/1 mL/via, 1 × 10^8^ cells/1 mL/vial	IM	VAS, PWD, ABI, TBI, UH, MTD	6	20
26	NCT04746599	NA/recruiting	Ad-MSCs	NA	NA	IA	NRS, ABI, tcpO_2_, AFS	6	20
27	NCT05475418	NA/not yet recruiting	Adipose tissue-derived exosomes mixed with hydrogel	Texas grade 1A–D, 2A–D	NA	Wound surface	UH	1	5
28	NCT01558908	I, II/unknown	ERC	Rutherford 4–5	25 × 10^6^, 50 × 10^6^, 100 × 10^6^	IM	AEs, ABI, VAS, UH, tcpO_2_	13	15
29	NCT03267784	I, IIa/completed	ABCB5-positive MSCs	Wagner 1–2	2 × 10^6^/cm^2^ wound surface area	Wound surface	AEs, ABI, UH, NRS, AR	12	23
30	NCT01216865	I, II/unknown	UCB-MSCs	NA	50 × 10^6^	IM	Angiogenesis, ABI, UH, PWD, AR	6	50
31	NCT03994666	II/unknown	UCB-MSCs	NA	60 × 10^6^, 120 × 10^6^	IM	AEs, ABI, tcpO_2_, DR	12	30
32	NCT03423732	II, III/active, not recruiting	CardioCell (WJ-MSC)	Rutherford 4–5	30 × 10^6^	IM, IA	ABI, tcpO_2_, AFS, UFS	12	50
33	NCT01859117	I/completed	Cenplacel (PDA-002) (P-MSCs)	Wagner 1–2	3 × 10^6^ 10 × 10^6^ 30 × 10^6^ 100 × 10^6^	IM	AEs, UH, ABI, TBI	24	15
34	NCT00919958	I/completed	PLX-PAD (P-MSCs)	Rutherford 4–5	175 × 10^6^, 315 × 10^6^, 595 × 10^6^	IM	AEs, IR tumorigenesis, AS	24	15
35	NCT00951210	I/completed	PLX-PAD (P-MSCs)	Rutherford 4–5	280 × 10^6^	IM	AEs, DR, AR, IR, VAS	6	12
36	NCT01679990	II/completed	PLX-PAD (P-MSCs)	Rutherford 2–4	NA	IM	MWD	12	180
37	NCT03006770	III/active, not recruiting	PLX-PAD (P-MSCs)	Rutherford 5	300 × 10^6^	IM	MA, DR, NRS, UH	36	213
38	ChiCTR-ONC-16008732	I/completed	P-MSCs	NA	1 × 10^6^ cells/kg	IM	AEs, PWD, MRA, ABI, UH, AR	6	4
39	IRCT20210221050446N1	I, II_a_/active	P-MSCs	Rutherford 4–6	20 × 10^6^, 30 × 10^6^, 60 × 10^6^	IM	AR, AEs, ABI, DSA, IR, UH, MTD, PWD, NRS	6	9

*ABI* ankle-brachial index, *AEs* adverse events, *AFS* amputation-free survival, *AR* amputation rate, *ASM* angiographic score of MRA, *DR* death rate, *DSA* digital subtraction angiography, *ETT* exercise treadmill test, *IA* intra-arterial, *ICA* indocyanine angiography angiogram, *ILP* improvement of local perfusion transluminal angioplasty, *IM* intramuscular, *IR* immunological reaction, *IV* intravenous, *MACE* major adverse cardiac event, *MRA* magnetic resonance angiography, *MTD* maximum tolerable dose, *MWD* maximal walking distance, *NA* not applicable, *NRS* numeric rating scale, *PWD* pain-free walking distance, *ERC* endometrial regenerative cell (menstrual mesenchymal stem cells), *TBI* toe-brachial index, *TcPO*_*2*_ transcutaneous oxygen pressure, *TWD* total walking distance, *UFS* ulcer-free survival, *UH* ulcer healing, *VAS* visual analog scale, *WBFRS* Wong–Baker FACES pain rating score, *WJ-MSC* Wharton’s jelly mesenchymal stem cell

Numerous comparative clinical trials between two groups of diabetic patients have demonstrated that the transplantation of bone marrow mesenchymal stem cells **(**BM-MSC) might be better in terms of rest pain, painless walking time, ABI, TcO2, and angiogenesis score analysis than bone marrow-derived mononuclear stem cells (BM-MNCs) in advancing lower extremity blood circulation and decreasing the discomfort of diabetic and CLI patients [76–79].

CHAMP is an ongoing phase I/II clinical research that began in September 2017 and compares the therapeutic benefits of intramuscular injections of allogeneic MSCs and autogenous concentrated bone marrow aspirate (cBMA) in patients with no other treatment options for CLI (no-CLI). Moreover, this study was designed to better understand the biological mechanism of allogeneic MSCs in human tissue. Subjects experiencing rest pain or tissue loss undergoing BKA are randomized to receive either cBMA or MSC. At that time, BKA is conducted, and tissue is collected to assess proangiogenic MSC persistence, cytokine explanation, progenitor cell quantification, and histopathological description [80].

Clinical investigations on therapeutic angiogenesis using BM-MSCs collect a considerable amount of bone marrow, which is painful for patients and may require general anesthesia, increasing mortality. Ixmyelocel-T can circumvent these obstacles by expanding CD90+ mesenchymal stem/progenitor cells and CD14+ monocytic stem/progenitor cells through an automated, closed ex vivo culture system. This enables bone marrow aspiration without general anesthesia and may be more effective than fresh bone marrow. For example, in the RESTORE-CLI, a Phase II trial, patients with CLI obtained Ixmyelocel-T or a placebo. Compared to placebo administration, Ixmyelocel-T therapy significantly delays the onset of treatment failure, as measured by major amputation, mortality, doubling of the wound's total area from baseline, or the development of de novo gangrene. After Ixmyelocel-T therapy, the rate of amputation-free survival was increased, although the difference was insignificant [81–83].

A phase II clinical study has employed engraftment of a cell product, including endothelial progenitor cells (EPCs) and BM-MSCs (MESENDO) into critically ischemic limbs, effectively encouraging blood vessel repair and improving clinical outcomes. The cell doses employed in this investigation were lower than those used in their initial phase I study; nevertheless, the stem/progenitor (MSC to EPCs) cell ratio was always one. As a result, a repertoire of stem/progenitor cells significantly impacts clinical results instead of the actual quantity of administered cells. These findings revealed that adding MSCs to the cell product will help modulate and enhance MNCs' described effect on inducing angiogenesis in ischemic limbs. Besides EPCs, developing new blood vessels necessitates the simultaneous engagement of mural cells (pericytes and vascular smooth cells) and the provision of essential chemokines, growth factors, and extracellular matrix components. Therefore, administering a cell product, including both MSCs and EPCs, may be essential for enhancing angiogenesis and improving ischemic tissue regeneration. As of right now, it is unknown whether the clinical improvement is due to angiogenesis or an increase in collateral remodeling via MSC [84].

Although these trials provided promising findings, numerous significant restrictions have prohibited cell-based treatment from becoming a widely used treatment technique. First, isolating autologous cells necessitates a harvest operation performed under anesthesia, which can be dangerous for CLI patients who are already at increased risk for problems due to their advanced age and cardiovascular disease. Second, when compared to the controls, autologous stem cells from patients suffering from cardiovascular illness demonstrated poor neovascularization potential in preclinical models of stroke and myocardial infarction. Circulating BM-derived progenitor cells in CLI patients is inefficient and significantly lower than in control subjects due to extended pro-inflammatory stimuli [85]. Lastly, some research revealed that autologous BM-MSC transplantation in CLI patients may has a risk of karyotypic aberrations. It is still not entirely understood if the detected karyotype abnormalities demonstrate that patients' cells naturally have aneuploidy or are generated in cultures [86].

According to several studies, MSCs derived from varied sources are equal and may not consistently reach the same effectiveness or result. Therefore, ongoing crosstalk between the transplanted cells and host may influence MSC actions, thus changing MSC's future capacity [15]. Accordingly, allogeneic MSCs administration has numerous advantages versus autologous MSCs; first, because no harvesting operation is required in administering allogeneic MSCs, the pressure on the patient is significantly reduced [87]. Second, whereas MSCs derived from healthy donors display stability and coherence in their biological and practical characteristics, autologous MSCs obtained from patients suffering from inflammatory and/or degenerative diseases demonstrate variability in their biological and functional properties, bringing about damaging consequences for the host when they have dealt with host signals [88, 89]. MSC derived from individuals with atherosclerosis develop a pro-inflammatory secretome by the generation of inflammatory cytokines like IL6, IL8, and MCP1, reversing their naturally immunosuppressive properties [90]. Furthermore, the proangiogenic capability of the isolated allogeneic cell can be assessed before administration [87]. There is significant variability among isolated MSC from the donor, with around 25% of MSCs failing to produce more significant neovascularization than placebo [91]. This heterogeneity affects trial outcomes and therapeutic capacity when MSCs are used autologously. The allogeneic application allows for the best available donor isolates, reducing treatment response variability. Eventually, there will be a significant cost differential in therapy. When it comes to allogeneic MSC application, it is possible to expand MSCs from altruistic donors before the research and therefore employ them as an off-the-shelf cell product. This enables the therapy to be immediately accessible and authorizes batch-based rather than patient-based testing (pre-treatment), which reduces costs [87]. Due to such advantages, the use of allogeneic cells was considered.

Stempeucel^®^, marketed by Stempeutics, has received limited approval in India to treat Non-Atherosclerotic and Atherosclerotic Critical Limb Ischemia [92]. Gupta's team conducted a phase I/II study to assess the impact of administering 2 million cells/kg MSCs generated from allogeneic bone marrow (Stempeucel) in CLI participants. The incidence of adverse events (AEs) was identical in both arms (BM-MSC and placebo) and was not associated with stem cells (related to disease progression). The BM-MSC group showed improvement in efficacy indicators such as rest pain score, ABPI, and ankle pressure [69].

Given these positive outcomes, another Phase II clinical trial (NCT01484574) evaluating the safety and effectiveness of different doses of Stempeucel in patients with critical limb ischemia was completed in 2016. There are currently no published data.

In a multicenter, randomized, double-blind, placebo-controlled phase II/III clinical study (SAIL), 66 patients with no-options CLI were randomized to receive 150 × 10^6^ allogeneic BM-MSCs over 30 injection sites or placebo intramuscularly in the ischemic limb. If this study demonstrates the immunity and effectiveness of allogeneic BM-MSCs in no-option CLI patients, a larger multicenter clinical trial built on these data will be conducted in the future, confirming allogeneic BM-MSCs' efficacy in CLI [87].

- Adipose-derived mesenchymal stem cells (AD-MSCs)

Adipose tissue is abundant in the human body and is replaced consistently. Accordingly, this tissue is an excellent substitute for MSCs [93]. Adipose-derived stem cells (AD-MSCs) share several functional and morphological characteristics with BM-MSCs. AD-MSCs were found to produce high levels of MMP-3 and MMP-9 that can enhance VEGF release, leading AD-MSCs to develop effectively into endothelial cells [75]. It was demonstrated that models of hindlimb ischemia administered with AD-MSCs had a higher rate of blood circulation recovery and better limb salvage than those who received BM-MSCs [94]. AD-MSCs have been demonstrated to release various angiogenic growth factors, including HGF, VEGF, and SDF-1. SDF-1, in particular, is thought to be important in AD-MSC-mediated angiogenesis because it promotes EPC mobilization into ischemia foci [74]. Unlike BM-MSCs, AD-MSCs could be collected from a small proportion of human subcutaneous adipose tissue at a relatively high concentration using minimally invasive procedures such as liposuction or excision of subcutaneous adipose tissue. They expand quickly in culture. This rapid proliferation generates sufficient cells to treat large-volume lesions [3]. Lee et al. previously performed a pilot clinical study to assess the safety and effectiveness of autologous AD-MSCs grafted into ischemic limbs of Burger's disease and diabetic patients. The number of transplanted cells (3 × 10^8^) used in this study was approximately 0.01 of the number of transplanted cells used in MNC studies. Expanded AD-MSCs were administered intramuscularly into patients' ischemic muscles. They demonstrated that this cell-based treatment could be viable and efficient for improving blood flow in individuals with CLI. This study found that diabetes patients' AD-MSC (D-AD-MSC) includes lower MSC than healthy subjects, yet AD-MSC functions normally. However, unlike AD-MSC obtained from Burger's patients (B-AD-MSC), D-AD-MSC demonstrated an altered growth capacity. Muscle stem cells, endothelial progenitor cells, and MSC were all affected by the hyperglycemia-induced reduction of stem cell activities. Even with the reduced functionality of D-AD-MSC, implantation of these cells led to a considerable improvement of ischemia manifestations in three diabetic subjects. The upregulation of VEGF under hypoxic environment and the similarity in angiogenic paracrine cytokine expression between AD-MSC and normal AD-MSC account for these outcomes. The upregulation of VEGF under hypoxic environment and the similarity in angiogenic paracrine cytokine expression between AD-MSC and normal AD-MSC account for these outcomes [93]. Similarly, ACellDREAM, the first phase I clinical trial, indicated that autologous AD-MSCs had an excellent tolerance and stimulating effects on skin oxygenation and healing in participants with no-CLI. These cells are a promising BM-MSC replacement for two reasons. Initially, when compared to BM-MSCs, they have a greater angiogenic capacity. These benefits are partially achieved by their development into endothelial-like cells and paracrine functions. Secondly, because the number of AD-MSCs isolated from adipose tissue is greater than that of MSCs harvested from BM, a more considerable number of mesenchymal cells can be collected in a shorter duration from adipose tissue than from bone marrow [95]. The findings guide the design of ACellDREAM2—a phase II, multicenter study—started in 2019 (NCT03968198). The present clinical trial aims to confirm the effectiveness parameters such as new vessel formation, blood flow, ulcer healing, and pain reduction in 43 no-option CLI patients after receiving autologous AD-MSCs in their inferior ischemic limbs.

AD-MSCs derived from healthy donors had homogeneous and consistent qualities, but those separated from patients with degenerative and inflammatory diseases exhibited various biological and functional properties [88, 89, 96]. Other research utilizing MSCs derived from patients with diabetes reveals that the proliferation, differentiation, and angiogenic ability of endogenous cellular resources are altered by the hyperglycemic environment and other metabolic factors associated with diabetes, differentiation, and angiogenic ability [97–99].

In this regard, Soria B et al. designed the NOMA Trial to evaluate the safety, tolerability, and efficacy of the intramuscular administration of allogeneic AD-MSCs in 90 eligible patients with type 2 diabetes with critical lower limb ischemia and no possibility of revascularization in 3 parallel groups (placebos, low-dose AD-MSCs, and high-dose AD-MSCs) [72].

- Umbilical cord blood mesenchymal stem cells (UCB-MSCs)

The animal experiment shows that UCB-derived MSCs (UCB-MSCs) could directly regenerate arterioles and differentiate into endothelial cells in vitro. UCB-MSCs offer several benefits due to (1) the immaturity of newborn cells compared to adult cells and (2) the ability to prevent immune reactions that result in dysfunctional grafts. As cord blood-derived stem cells are less vulnerable to attack the recipient's body than bone marrow-derived stem cells, a new pattern for stem cell therapy without immunosuppressive drugs has been devised. Due to their high potential for ex vivo proliferation and differentiation, UCB-MSCs are potentially a valuable source for cellular treatment for vaso-occlusive disorders [100].

SW. Kim et al. conducted the first clinical trial of intramuscular administration of human leukocyte antigen-matched UCB-MSCs, involving four patients with thromboangiitis obliterans (TAO). They demonstrated that arterial reconstruction or prevention of arterial obstruction using UCB-MSCs directly controls rest pain and accelerates the healing process of ischemic ulcers. Intriguingly, all treated patients had a rapid improvement in rest pain prior to vascular formation. Researchers believe that growth factors or pain releasers secreted by implanted stem cells before vessel generation in ischemic regions may be responsible for the pain relief [100]. Based on the results of previous study, another clinical experiment was designed to evaluate the safety and feasibility of intramuscular transplantation of UCB-MSCs into the affected limbs of patients with atherosclerosis obliterans (ASO) or TAO. Angiographic scores improved in three of eight patients and complete ulcer healing. Although all patients had rising digital subtraction angiography scores, three developed collateral arteries in the injected limbs. This research established that intramuscular injection of UCB-MSCs is a safe and well-tolerated therapeutic option for individuals with end-stage CLI [101].

In another clinical trial, 24 patients were injected with UCB-MSCs. Comparing clinical improvement and neovasculogenesis at baseline and six months after UCB-MSC injection demonstrated considerable improvement in the patients' primary symptoms, including rest pain, pain-free walking distance, and ulcers. CT angiography revealed the creation of new collateral arteries; this alteration was more apparent in the microvascular network than in the macrovascular network. Furthermore, compared to pre-treatment levels, the percentages of CD3+ CD8+ lymphocytes were dramatically raised following treatment with UCB-MSCs, while percentages of CD3+ CD4+ lymphocytes and CD3-CD16/CD56+ NK cells were significantly reduced [102].

- Placenta-derived mesenchymal stem cells (P-MSCs)

Placenta-derived mesenchymal stem cells (P-MSCs) are derived from the extraembryonic mesoderm and exhibit the same morphological characteristics as other MSCs [103]. They are capable of trilineage differentiation, maintaining long telomeres, and expressing pluripotency markers such as Oct4 and SSEA-3 [104]. In this context, P-MSCs exhibit remarkable biological features that can be maintained in a GMP-grade (good manufacturing practice) culture environment, and they can be widely utilized in translational medicine;

1. *Immunomodulatory characteristics* P-MSCs have a greater immunomodulatory capacity than UCB-MSCs [42]. P-MSCs, like other MSCs, express low levels of HLA-A, HLA-B, and HLA-C, making them less immunogenic and hence more appropriate for transplant. P-MSCs promote the transformation of pro-inflammatory M1 cells into anti-inflammatory M2 cells. Additionally, PM-MSCs express HLA-G, inhibiting T cell growth [31]. A comparative investigation of UCB-MSCs and P-MSCs on mononuclear cells and dendritic cells revealed that P-MSC conditioned medium (CM) inhibited T cell proliferation significantly more than UCB-MSC, signifying that cell-to-cell contact is unnecessary [105]. Although few details on the P-MSC interaction with B cells are known, there is evidence that it prevents B cells from undergoing early apoptosis. P-MSCs also have immunosuppressive effects on natural killer (NK) cells [106].
2. *Proliferative and clonogenic ability* It has been established that P-MSCs are more durable, proliferative, and exhibit more long-term potential for growth than BM-MSCs [107, 108]. P-MSCs can also be interpreted as a reflection of the cell's ability to develop high density, which is considered a hallmark of a stromal cell [60]. A comparative investigation of UCB-MSCs revealed that P-MSCs formed more colonies than UCB-MSCs isolated from the same tissue. This may also be since P-MSCs have a greater capacity for expansion [105].
3. *The anatomical position* The placenta, a structure with high vascularity, is subjected to elevated amounts of angiogenesis stimulating signals, creating an ideal microenvironment for angiogenesis-promoting signals. Additionally, studies have demonstrated that P-MSCs are predominantly found in the placenta's vascular niche [109].
4. *A significant source of fetal stem cells* Although ethical constraints prohibit embryonic stem cell utilization, fetal stem cells have been suggested as a substitute. Fetal stem cells are more primitive and robust than adult stem cells, making their usage in medical applications more effective. The placenta is an excellent source of fetal stem cells, which exist in a state between adult and embryonic stem cells [110].

P-MSCs were the sort to be harvested as a therapeutic product, validating the feasibility of a placenta-isolated "mass-produced" source of therapeutic cells. Their initial findings indicated that intramuscular delivery of P-MSCs in a BALB/c mice model of hindlimb ischemia resulted in decreased endothelial dysfunction and improved limb function [111]. Additional phase I through phase III clinical studies employing P-MSCs given by intramuscular injections for critical limb ischemia are underway, with promising results (Table 3).

Wu et al. investigate the safety of P-MSCs (PDA-002) in patients with PAD and DFUs in phase I clinical research. To estimate the MTD or the highest planned dose level, they designed a 3 + 3 dose-escalation strategy (3 × 10^6^, 10 × 10^6^, 30 × 10^6^, and 100 × 10^6^ cells). After administration of P-MSCs, there was initial evidence of wound repair, enhanced peripheral circulation, and decreased levels of vascular injury biomarkers (circulating ECs level). P-MSCs were typically safe and effective for chronic DFU and PAD patients [112].

PLX-PAD is an "off the shelf" placental-derived mesenchymal like cell product that has exhibited anti-inflammatory, proangiogenic, and regenerative effects in preclinical investigations. While PLX-PAD cells show surface markers expected of conventional mesenchymal stromal cells, their ability to develop in vitro into cells of the mesodermal lineage is low. As a result, their suggested mechanism of action is the timely release of different proteins that stimulate immunomodulatory properties, angiogenesis, and support muscle tissue regeneration. PLX-PAD cells have been proven in vitro to increase endothelial cell proliferation [113]. Proangiogenic proteins such as VEGF, HGF MMP-1, MMP-2, and angiogenin are secreted by the cells and are increased in hypoxic cultures [113, 114]. Angiogenin then interacts with SMCs and ECs, causing cell proliferation, migration, tubular structure development, and invasion [115]. Muscle regeneration may be beneficial in CLI since ischemia circumstances cause muscle atrophy. PLX-PAD cells have been demonstrated to increase skeletal muscle cell migration in vitro, speed muscle regeneration, and recover muscle activity in vivo. These studies have also revealed that PLX-PAD cells injected locally do not disperse to other tissues, validating the proposed mechanism of action of PLX-PAD cells-mediated protein secretion [11].

Two phase I trials were done to determine the safety of PLX-PAD cell intramuscular injections in CLI subjects who were not eligible for revascularization. In Study 1202-1, Doses of 175 million cells, 315 million cells, and 595 million cells were administered in three separate doses. Study 1202-2 compared a single dose of 280 million cells to two doses of 280 million cells (2 weeks apart). In general, the therapy was safe for patients with CLI, and HLA matching was shown to be unnecessary. Because the number of patients in this study was not so large to demonstrate clinical benefit, several variables have suggested a significant medical effect. The pooled amputation-free survival rates at six months and one year were higher than those recorded in identical patient groups. In all research groups, pain scores declined after treatment with PLX-PAD, while TcPO2 showed an increasing tendency during the time [11].

PACE is a phase 3 multinational placebo-controlled parallel-group trial including 246 patients with atherosclerotic CLI. Its purpose is to evaluate the safety and effectiveness of intramuscular administration of 300 × 10^6^ PLX-PAD cells. Two cell administrations, rather than one, have been demonstrated to be more effective in hindlimb ischemia models and human subjects; hence the study design includes a second administration procedure two months following the first. After the PACE trial is completed, a conclusion can be made on the study's outcomes and the efficacy of PLX-PAD cells [11].

Nevertheless, these studies do not assess P-MSCs administration outcomes in non-atherosclerotic PAD (Berger’s disease). Berger’s disease is a non-atherosclerotic inflammatory disease of unknown etiology. It has a worldwide distribution and is more prevalent in the Middle East and the Far East than in North America and Western Europe [116]. Recently we conducted a dose-escalation study phase I/IIa (IRCT ID: IRCT20210221050446N1) that aimed to assess the safety and efficacy of intramuscular delivery of P-MSCs injection in 9 atherosclerotic and non-atherosclerotic PAD patients with CLI. 6 months after P-MSCs transplantation there were remarkable improvements in the target limbs such as rising angiography scores, less rest pain, ulcer healing, increase in pain-free walking distance (to be published).

These findings demonstrate that MSCs derived from alternate sources can rival with BM-derived stem cells because of less invasive extraction approaches. Although this therapy has improved subjective and objective perfusion properties, no significant results in amputation rate reduction have been obtained. More clinical trials will be required to determine the MScs' clinical efficacy in patients with PAD/CLI.

- MSCs derived from human pluripotent stem cells (hPSC-MSCs)

Collecting MSCs from different tissues has several drawbacks, including restricted cell proliferative potential and limited supply in the tissues, which results in insufficient MSCs; gradual decline of cell differentiation throughout in vitro expansion, which lessens the efficacy of MSC administration; and variability between donors (state of health, tissue origin, and donor age), which intensifies the variability of MSC effectiveness, leading to impeded therapeutic applications of MSCs [117–119]. Even though MSCs can be taken from many different types of tissues, almost all of them go through the senescence process, which reduces their ability to both proliferative potential and differentiation ability. Therefore, pluripotent stem cells, specifically induced pluripotent stem cells (iPSCs), have long been of attention as a potential source for MSCs [120]. hPSC-MSCs or MSCs produced from human pluripotent stem cells can bypass these difficulties related to tissue-derived MSCs. iPSC-MSCs have similar antigen, gene expression, and epigenetic profiles as MSCs. iPSC-MSCs are differentiated into three lineages, identical to BM-MSCs [119]. When triggered with IFN-γ, iPSC-MSCs, like BM-MSCs and fetal-MSCs (fMSCs), express the lowest level of HLA-II. YQ Sun.et al. evaluated the regeneration effectiveness, inflammation, cell persistence, and HLA II expression of human MSCs generated from iPSCs, fetuses (fMSCs), and adult bone marrow (BM) in immunological humanized NOD Scid gamma (NSG) mice with hindlimb ischemia after IFN-γ stimulation. Transplantation of iPSC-MSCs resulted in more significant recovery from hind limb ischemia and higher cell survival rates than BM-MSCs. These results demonstrate that iPSC-MSCs are immunologically privileged upon transplantation and resistant to IFN-γ activation. They may have greater therapeutic implications and a lower probability of allogenic transplantation rejection [121]. hPSC-MSCs may represent the most encouraging patient-specific cell source for stem cell therapy due to their unlimited self-renewal and differentiation capacity toward MSCs [122]. iPSC-MSCs surpassed BM-MSCs in reducing severe hindlimb ischemia, which could be attributed to greater in vivo survival and trophic elements and increased iPSC-MSC proliferation [123]. The higher survival and engraftment following administration of iPSC-MSCs may be due to their increased ability to stimulate muscle and vascular recovery through paracrine pathways and straight de novo differentiation. Human induced pluripotent stem cells (hPSCs) can be used to clonally create MSCs with the ability to perform various functions. Tissue ischemia can be treated with patient-specific iPSC-MSCs generated in an "off-the-shelf" fashion [124].

Concerns about employing iPSCs have always included the potential of teratoma development attributed to cell populations and cancer formation in iPSCs due to their higher genetic and epigenetic instability [176]. Strict QA/QC techniques which exclude undifferentiated and genetically unstable cells can alleviate these problems [120].

Differentiation ability is an essential characteristic of hPSC-MSCs for therapeutic use, yet very little is known about the various properties between hPSC-MSCs of different sources. Variations in iPSCs can be attributed to insufficient reprogramming or epigenetic memory; indeed, iPSC-MSCs show preferred differentiation into their initial lineage cells. However, various therapeutic benefits of iPSC-MSCs from various sources were reported, implying that the effectiveness of hPSC-MSCs is cell origin dependent. Furthermore, the quality and effectiveness of hPSC-MSCs produced through differentiation techniques obviously vary [119].

Two phase 1/2 clinical trials involving iPSC-MSCs are now filed, one for GvDH in 2016 and another for COVID-19/ARDS in 2020. An interesting new milestone is the publication of the GVHD test outcomes, which show that the treatment is safe and has some promising signs of effectiveness [125]. Additional clinical studies utilizing hPSC-derived MSCs are expected to be conducted due to growing preclinical results and these sentinel clinical studies [120].

In 2018, a notable observational clinical trial (NCT03403699) employing MSCs produced from iPSCs examines if iPSCs can be utilized efficiently as a potential treatment option for diabetic retinal ischemia. iPSCs will be produced from the peripheral blood cells of diabetics and age-matched controls. Human iPSC will be employed to develop mesoderm cells for injection into diabetic rodent and primate eyes' vitreous cavities. In areas of degenerated capillaries, the ability of mesoderm cells to develop pericytes and ECs will be investigated. Human iPSCs will also be employed to derive CD34+CD45+ hematopoietic cells. The combination of CD34+CD45+ cells generated from iPSCs and iPSC-derived mesoderm will be tested for their potential benefit in enhancing vascular development. These paradigm-shifting and novel observations show that the hiPSC-derived-mesoderm subset can be used for long-term revascularization of vasodegenerative diseases such as CLI because their tissue-repair action can be augmented further by mixed utilize of CD34+CD45+ cells, which supply anti-oxidant and anti-inflammatory effects [126].

### Biocompatible scaffolds for CLI treatment

As stated above, MSC therapy may be a potential therapeutic method for therapy of CLI patients, given that MSC can persist and engraft within the ischemic region. Engineered scaffolds are designed to enhance the survival rate of injected MSCs and provide physical support for cell adhesion. They create an appropriate environment for delivery by harmonizing and sustaining cell connections to promote vascularization and angiogenesis after implantation [127–129]. Scaffolds can be either natural or synthetic biomaterials, with synthetic biomaterials being more manageable due to their high degree of tissue compatibility [127]. For example, Carrabba et al. constructed channel-shaped scaffolds using the synthetic polymer polycaprolactone and then deposited gelatine nanofibers. These scaffolds were subsequently placed around the femoral artery of CLI-induced animals and seeded with adventitial progenitor cells (a source of smooth muscle cells). The study's findings indicated improved blood flow and arteriogenesis in CLI-induced animals and proposed that this synthetic scaffold represents an innovative and promising strategy for targeted administration of therapeutic cells in CLI patients [130].

Hydrogel application while administering stem cells to improve wound healing in DFUs encountered some difficulties, including low cell retention and stem cell integration. According to Shi et al. [131], gelatin microspheres increase the delivery and integration of rat adipose-derived stem cells administered locally. Results suggested that gelatin microspheres effectively promote healing in DFUs by increasing M2 macrophage differentiation, angiogenesis, peripheral nerve regeneration, and collagen deposition. In a separate study, Takahashi et al. [132] found that the application of hydrogels enclosing conditioned media of amnion-derived MSCs cultured under hypoxia enhances wound healing in diabetic mice by promoting angiogenesis, impairing inflammation, and accelerating epithelization. This study's findings remove the need for local stem cell transplantation while further investigation is necessary.

Laiva et al. developed a novel collagen-chondroitin sulfate scaffold that works with polyplex nanoparticles containing the gene encoding for stromal-derived factor-1 alpha (SDF-1 gene-activated scaffold). They evaluated the effect of the gene-activated scaffold on diabetic AD-MSCs through capering response of diabetic AD-MSCs to that of healthy AD-MSCs cultured on a gene-free scaffold. They discovered that the gene-activated scaffold could recover the proangiogenic response in human diabetic AD-MSCs identical to the gene-free scaffold in healthy AD-MSCs. Transfected diabetes patients' AD-MSCs further demonstrated pro-wound healing characteristics such as active matrix remodeling of the fibronectin matrix and basement membrane protein collagen IV. They demonstrate that the SDF-1 gene-activated scaffold may overcome the limitations of diabetic AD-MSCs, creating opportunities for stem cell therapies in combination with new biomaterials to treat DFUs [133].

Preclinical trials using biomaterials for CLI therapies have shown promising outcomes, but more research is needed before they can be used clinically. Apart from the ability to promote the formation of blood vessels and maintain the transplanted cells, different chemical and physical features of scaffolds should be investigated before they are employed in clinics. Moreover, it has been mentioned that regulatory issues related to FDA, delivery methods throughout product development, and manufacturing approaches are the most significant parameters when designing a biomaterial. Therefore, addressing these issues may enhance their therapeutic potential and treatment outcomes for individuals with CLI [10].

In a case study, Zeng et al. evaluated a 57-year-old patient with type 2 diabetes and a 20-day DFU receiving P-MSCs hydrogel (1 × 10^6^/cells/cm^2^) in the wound to examine the efficacy and safety of P-MSCs hydrogel in wound repair. The foot ulcer had nearly healed, granulation-tissue production had increased, and lower extremity amputation had been successfully avoided. In the six months following, no adverse events were detected. According to this case study, P-MSCs hydrogel may offer a novel strategy for DFU treatment [134].

### MSC secretome-mediated angiogenesis

Recent research on MSCs transplantation as a cell-based treatment in patients with CLI indicates that the release of biologically active molecules such as growth factors, cytokines and chemokines, angiogenic factors, ECM proteins and proteases, and genetic material secreted from stem cells for cell communication, known as secretome, may be the key to successful cell therapy [68, 135]. Secretomes have also been referred to as "conditioned medium" (CM) in many studies due to their ability to interfere with various biological activities, including proliferation, division, differentiation, apoptosis, and signal transduction. The secretome of stem cells has revealed considerable capacity and allows for the repair and regeneration of damaged cell membranes or induces the secretion of surrounding tissues [136]. Secretomes generated from the various progenitor, or stem cells are being investigated, mainly using mass spectrometry techniques. Barberg et al. evaluated the secretome composition of MSCs in this manner, indicating proteins involved in cell proliferation, signaling, and communication, along with growth factors and cytokines involved in hematopoiesis physiological control [137].

Similarly, Maffioli et al. demonstrated that MSCs enhance the release of proteins involved in immunomodulation and angiogenesis in a pro-inflammatory environment [138]. A complete understanding of the functions and determinants of the secretomes would enable us to reconstruct them using bioactive molecules for tissue regeneration [12]. Given that stem cell treatment may sometimes encounter ethical obstacles or biological restrictions, using secretomes as a substitute for cell therapy overcomes potential disadvantages such as immune rejection or tumorigenesis [136]. One of the crucial approaches mediated by the MSC secretome is through microvesicles and exosomes, which are known to regulate the physiological processes mediating angiogenesis [37]. Exosomes are nanometer-sized, membrane-bound vesicles containing lipid bilayer-encapsulated paracrine elements. MSC exosomes contain particular microRNAs (miRNAs) that promote angiogenic and regenerative processes that target specific cells, specific gene expression, and signaling pathways, which targeted cells can internalize to promote signaling pathways [3, 139]. In conclusion, MSC exosomes are thought to have identical biological properties to MSCs. Exosome therapy derived from MSCs appears as a promising therapeutic approach for treating various illnesses, including limb ischemia [3]. Numerous studies implicated the role of miRNAs in capillary repair, angiogenesis, and cardioprotection, including miR320, 132, 21a-5p, and 126, as well as the miR-27b, miR-17-92 cluster, miR210 [140], and mir-130a [141, 142]. However, some publications demonstrate that miRNA125a (adipose-derived exosome) promotes angiogenesis by suppressing the angiogenic inhibitor delta-like 4 (DLL4) in endothelial cells [143]. By establishing contact between ECs and MSCs under conditions of in vitro coculture, miRNAs generated from MSCs may exhibit angiogenic activities. The development of gap junctions enables the translocation of miR200b toward the endothelium where by targeting GATA2, ZEB2, KDR, and VEGF, suppresses angiogenesis [144], implying that particular miRNAs influence both MSC and tissue fate in the microenvironment. Therefore, MSC-derived exosomes' synthesis, relative stability, and composition vary with the environment and vascular injury. For example, miR-21a-5p seems to be the most common regulator inhibiting pro-apoptotic gene products such as FasL, Peli1, PDCD4, and PTEN, in host cardiac cells after in vivo myocardial infarction [145]. Similarly, the microenvironment may influence miRNA activity. Exosomes derived from MSCs are highly sensitive to hypoxia, controlling the VEGF/VEGFR pivot in the host tissue or, more precisely, the VEGF concentration of the exosomes. There is a lack of clarity on the biochemical signaling pathways, content, subpopulations, and various biological activities of extracellular vesicles derived from MSCs [37]. Consequently, since exosomes are very heterogeneous, the importance of the cell niche and the techniques utilized are being thoroughly investigated. More research is needed to investigate this, and if prosperous, these findings could reveal a new occasion for the cell-free treatment [146].

Preclinical results on cell-free products produced from hPSC-MSCs have also recently appeared, with therapeutic immune results observed [147]. Hu et al. reported that transplantation of hPSC-MSCs-derived exosomes could ameliorate hindlimb ischemia injury in a mouse model. HPSC-MSCs-Exo can stimulate angiogenesis in ischemic tissue and increase vascular perfusion in the ischemic muscle, which are both essential for functional limb recovery. In addition, this work indicated that hPSC-MSCs-Exo can induce the migration, proliferation, and tube formation of human umbilical vein endothelial cells (HUVEC). Furthermore, hPSC-MSCs-Exo can stimulate the expression of molecules involved in angiogenesis in HUVECs. These data suggest that hPSC-MSCs-Exo could promote angiogenesis to reduce the severity of ischemia injury in the hindlimb animal model [148].

## Challenges

In most clinical studies performed, patients receiving MSC-based treatment demonstrated improved clinical symptoms and tolerance [37]. Despite these encouraging findings, several remaining concerns and challenges emphasize the need for preclinical studies to extend the period of animal trials to prove the regenerated micro/macro capillaries' long-term patency [149]. Moreover, well-designed cell therapy with patient numbers enough to enable statistical analysis should be considered [7]. Apart from exploring the specific molecular pathways supporting stem cell therapy's beneficial effects, there is much to learn about this novel treatment approach [9].

- The most efficient administration route of MSCs

While new research suggests that MSCs enhance collateral vessel development in individuals suffering from severe PAD, considerations about the best form of administration must be regarded [9]. As indicated in Table 3 most therapeutic trials addressing PAD have focused on intramuscular cell administration as a more feasible and less harmful approach [15]. Intramuscular injection is based on generating a cell depot with paracrine activity in the ischemic site (into the gastrocnemius muscle) [150]. The intravascular route, which is more invasive and more harmful, involves the administration of IV contrast, which is not recommended in chronic renal disease patients [151]. Systemic delivery, such as intravenous (IV) or intra-arterial (IA) infusion, is used less frequently [152].

Animal investigations have shown many entrapment and embolus development in the lungs after systemic intravenous injection [153]. Moreover, intravenously administered MSCs inhibit endothelial cell proliferation and angiogenesis via cell–cell contact through modulation of the VE-Cadherin/β-catenin signaling pathways [154]. MSCs express procoagulant activity (PCA) linked to the expression of tissue factor (TF) that, when in contact with blood, initiates coagulation. Several preclinical and clinical studies reported thrombogenic event during intravenous MSC infusion [155–158].

Intra-arterial injection entails the danger of injury to the nerves and arteries, vessel wall dissection, and dislodgment of atherosclerotic plaques [87]. The intramuscular injection, studied more extensively, is easy to use, less invasive, and has demonstrated safety and effectiveness [151]. This delivery approach brings about a temporary cell placement in ischemic areas, while the intra-arterial injection is targeted to effectively cell transfer to peri-ischemic regions that are assumed to provide enough oxygen and nutrients to conduct cellular functions [15]. One study compared the safety and efficacy of single intramuscular BM cell therapy (*n* = 15) to combination intra-arterial plus intramuscular BM cell delivery (*n* = 12) in patients who were not surgical or endovascular candidates [159]. While this research found no statistically significant difference between the two approaches, it could not distinguish between ABI, ulcer healing, and walking distance advancement. ABI and TcPO2 levels increased after intramuscular cell administration but not following intra-arterial cell treatment. Both significantly improved rest pain and pain-free walking distance, even though there was no distinction between them [160].

A comparative clinical trial comparing intra-arterial or intra-arterial plus intramuscular cell administration revealed that intramuscular cell therapy improved ulcer healing dramatically; In contrast, intra-arterial cell therapy trials could not be evaluated in detail. The ABI and TcPO2 were observed to improve considerably only when intramuscular or combination treatment was used but not when intra-arterial cell therapy was used alone [161, 162]. Based on such obvious facts, the authors believe that the intramuscular route should be the preferred route of administration [151].

- Development of a functional ischemic disease model relevant to CLI patients

Cell-based treatments were established to translate laboratory-based technologies into successful clinical treatment. As is frequently the case with preclinical research, encouraging animal model findings may not always interpret into promising clinical trials. One probable explanation is that experimental animal models are inherently incapable of accurately modeling human diseases [149]. The surgical model of hindlimb ischemia in immunodeficient mice created by femoral artery ligation was used to demonstrate the effectiveness of human cell populations transferred. However, surgical resection and chronic occlusion in atherosclerosis have few similarities [7].

Regarding limb ischemia modeling, rodents’ blood flow rates are very different from humans [149]. Whereas angiogenesis may be adequate to increase blood flow in the tiny tissue of the hindlimb mouse, the much bigger human leg may require broader channels to create adequate blood circulation [7]. Lastly, the majority of preclinical trials were conducted in two weeks. Therapeutic angiogenesis aims to develop a permanent neovasculature capable of providing constant blood perfusion to ischemic regions. Although, the majority of experiments are not successful in proving the ongoing de novo vessel formation and not retrogressing when therapy is terminated [149]. As a result, optimized transplantation experimental models that incorporate hyperglycemia, chronic inflammation, and hyperlipidemia comorbidities are required for a more accurate evaluation of cell transplantation before clinical trials [7]. Ultimately, one significant barrier is the absence of a practical human in vitro ischemic model in which studies on the specifics and effectiveness of MSC administration can be readily conducted. Three-dimensional tissue models allow for the systematic and repeated study of tissue or cell physiology, are cost-effective and less time-consuming, enable high-throughput evaluation, and can be applied similarly between research centers. This could help accelerate the interpretation of encouraging laboratory results for medical usage [163].

- Selection of appropriate patient populations for stem cell transplantation

CLI has a variety of causative agents, and ASO patients account for the predominance of patients with CLI [164]. There is considerable variation in their capacity to develop significant vascular repair when evaluating arteriogenic bypass mechanisms in individuals with PAD. The severity of perfusion impairment in individuals with equal degrees of arterial blockage can differ extensively [7]. Additionally, the severity of ischemia in patients chosen for cellular treatment should be evaluated. Walter et al. found that individuals with Rutherford stage 6 did not respond to cellular treatment, although those with stage 4–5 did [165]. Autologous strategies could be more advantageous in the early stages of the disease, such as in individuals with intermittent claudication (IC), where cell transfer may substantially impact the preservation of regeneration capacity by both injected and cells derived from the recipient [166]. On the other hand, as previously noted, concurrent patient diseases or atherogenic risk factors often alter either stem cell quantity or functions. These aspects must be clarified before employing cell therapy for therapeutic angiogenesis [167].

- Isolation, proliferation, and transplantation of MSC with complementary activities

Another constraint is that MSC for fundamental studies and clinical applications is generated as specific cell products in various facilities, often using various expansion media. Indeed, the various conditioned media modify the immunomodulatory properties of MSC [168]. Standard culturing procedures must be created, evaluated, and applied to ensure compliance with regulatory requirements, the achievement of safe and repeatable findings in a clinical context, and the translational potential of the results [169, 170]. Because MSC characteristics are not standardized, there is little agreement on whether MSC features benefit specific outcomes [37]. MSC cell populations have been extracted from various tissues and expanded in culture utilizing nonstandard protocols. Additionally, several reported research used cells exposed to serum derived from animals [75].

Bioequivalence across cell products and batches must be extensively studied to account for the variability of phenotypes among various MSC products to find those with tremendous therapeutic benefits and guarantee their safety [37]. Bioengineering techniques like 'fed-batch' automatically generated systems and enormous bioreactors have been designed. For progenitor cells' robust, safe, and cost-effective proliferation [171, 172]. However, it is well established that prolonged culture has a detrimental effect on regenerative capability. Lastly, ex vivo culture creates an opportunity for modulating stem cell activity [173]. In fact, during the culture process, CLI-induced dysfunction in MSC can be modulated [91]. Increasing the cell potency throughout the culture is one way to revitalize cells prior to administration [174]. Preconditioning techniques, on the other hand, have emerged to enhance the differentiation and immunomodulatory capacity of MSCs. Because physiological microenvironments are commonly hypoxic, MSCs cultured under such environments have increased survival and expression of cytoprotective chemicals. It has been shown that preconditioning MSCs with cytokines like IFN-γ or TNF-α increases their release of immunomodulatory factors.; however, these effects are not permanent [29]. Another point of contention is the cell dosage to apply, which varies according to the cell type/source [12]. The ideal number of cells to employ for angiogenesis is unknown. For example, in BM-MNCs, excessive cell injection resulted in adverse effects in animal models [175]. There have been limited studies on cell dose, and such research will be critical to optimizing the administration of expanded stem cell populations or marker-selected.

- Tumorigenesis possibility

Despite MSCs' anti-inflammatory and immunomodulatory properties, there are several growing concerns about their possible tumorigenicity due to their natural tendency to homing to damaged tissue and inflammatory areas [176]. In this respect, the existing microenvironment might impact the actions of MSCs, leading them to develop supportive features for cancer cells. No cancer has yet been discovered or recurred in clinical trials due to therapeutically administered MSCs. Nevertheless, potential risks associated with the growth and expansion of resident or undetected cancer cells are found in the body, or "resident" cancer cells persist. In conclusion, additional research is needed to determine whether MSC has the potential for tumorigenicity through the administration of MSC-based therapies [177].

## Conclusion and future perspective

Given the prevalence of CLI and its complications, such as amputation and vascular issues in no-option patients, therapeutic angiogenesis is a vital technique for increasing blood flow to ischemic regions. MSC-based therapy is regarded as one of the most encouraging cells for establishing angiogenesis in treating CLI subjects, including restoring tissue function and repairing ischemic tissue via immunomodulation, angiogenesis, and paracrine secretion of bioactive factors.

Overall, we have covered clinical trials using different sources of MSCs for treating patients with CLI. According to research on MSC-based therapy, the recent use of MSCs derived from alternate sources such as adipose tissue, umbilical cord blood, and placenta can rival BM-derived stem cells because of less invasive extraction approaches. Nevertheless, several remaining concerns related to the application of MSCs, including the development of a functional ischemic disease model relevant to CLI patients, appropriate patient populations for stem cell transplantation, isolation and proliferation protocols of MSCs, administration route and tumorigenesis possibility of these cells, emphasize the need for further investigations to optimize their use in personalized regenerative medicine by conducting more clinical trial studies. Therefore, there is much more to discover about this innovative therapeutic strategy.

## Data Availability

Not applicable.

## Abbreviations

ABI: Ankle-brachial index
AD-MSCs: Adipose-derived stem cells
ADT: Adipose tissue
AEs: Adverse events
AFS: Amputation-free survival
AR: Amputation rate
ASM: Angiographic score of MRA
ASO: Atherosclerosis obliterans
BM: Bone marrow
BM-MNCs: Bone marrow-derived mononuclear stem cells
B-cell: B lymphocyte
CLI: Critical limb ischemia
CM: Conditioned medium
DFUs: Diabetic foot ulcer
DR: Death rate
DSA: Digital subtraction angiography
ECM: Extracellular matrix
ECs: Endothelial cells
EPCs: Endothelial progenitor cells
ETT: Exercise treadmill test
FGF: Fibroblast growth factor
GATA2: GATA-binding factor 2
G-CSF: Granulocyte colony-stimulating factor
HGF: Hepatocyte growth factor
hPSC-MSCs: MSCs derived from human pluripotent stem cells
HUVEC: Human umbilical vein endothelial cells
IA: Intra-arterial
ICA: Indocyanine angiography angiogram
ICAM-2: Intercellular adhesion molecule 2
IC: Intermittent claudication
IDC: Immature dendritic cell
IL: Interleukin
ILP: Improvement of local perfusion transluminal angioplasty
IM: Intramuscular
INFγ: Interferon gamma
IR: Immunological reaction
LFA-3: Function-associated antigen 3
IV: Intravenous
MACE: Major adverse cardiac event
MCSF: Macrophage colony-stimulating factor
mDC: Mature dendritic cell
MHC: Major histocompatibility complex
MMPs: Matrix metalloproteinase
MO: Monocyte
MQ-M_2_: Macrophage M_2_
MRA: Magnetic resonance angiography
MSC: Mesenchymal stem cells
MTD: Maximum tolerable dose
MWD: Maximal walking distance
NA: Not applicable
NK: Natural killer cells
NKG2D: Natural killer group 2 member D
NRS: Numeric rating scale
PAD: Peripheral arterial disease
PB: Peripheral blood
PDCD4: Programmed Cell Death 4
PDGF: Platelet-derived growth factor
PD-L1: Programmed cell death 1 ligand 1
PELI1: Pellino E3 Ubiquitin Protein Ligase 1
PFWD: Pain-free walking distance
PGE2: Prostaglandin E_2_
PI3K: Phosphatidylinositol-3-kinase
PTEN: Phosphatase and tensin homolog
RPS: Rest pain score
SMCs: Smooth muscle cells
TAO: Thromboangiitis obliterans
TBI: Toe-brachial index
TcPO2: Transcutaneous oxygen pressure
TGFβ: Transforming growth factor beta
TNFα: Tumor necrosis factor alpha
TNT: Tunneling nanotube
T cell: T lymphocyte
TSG-6: TNF-stimulating gene-6
T-reg: Regulatory T cell
UCB: Umbilical cord blood
UCB-MSCs: UCB-derived MSCs
UH: Ulcer healing
VAS: Visual analog scale
VCAM-1: Vascular cell adhesion protein 1
VEGF: Vascular endothelial growth factor
WBFRS: Wong–Baker FACES pain rating score
ZEB2: Zinc Finger E-Box Binding Homeobox 2
